# Results of the Employment-Activity Program (EAP) of the PEPsNA in patients with first-episode psychosis

**DOI:** 10.1192/j.eurpsy.2022.1992

**Published:** 2022-09-01

**Authors:** L. Aranguren, A. Aquerreta Unzue, M.C. Ariz Cia, L. Azcarate Jimenez, A. Fernandez Falces

**Affiliations:** 1 Osasunbidea, Primeros Episoidos Psicóticos, Pamplona, Spain; 2 Osasunbidea - Mental Health, Primeros Episodios Psicóticos, Pamplona, Spain

**Keywords:** First-episode psychosis, Employment-Activity Program, Results

## Abstract

**Introduction:**

The Employment-Activity Program (EAP) of the PEPsNA aims that people develop or regain the occupational roles to wich ther aspire in their life after a first episode, due to the early ages affected by psychosis. Social-labor integration strategies (vocational rehabilitation and supported employment) are effective tools to improve the evolution of people with serious mental illnes.

**Objectives:**

Check the objectives achieved by the PEA, which in turn serves as a self-assessment, in order to improve our daily work.

**Methods:**

Data related to employment/occupation are analyzed, (number of people who are active in employment or carrying out standarized studies, people with temporary labor disability or who repeat a year, sheltered employment, occupational center, long-term unemployment and others), instrumental functional capacity and neurocognitive status (CGI COG scale), occupational disability (WHODAS scale), social and occupational functioning (SOFAS scale) and quality of life (QLS scale)

**Results:**

The rate of active persons with employment or standarized studies increases by 7.2% at 24 months, and the long-term unemployment rate decreases by 4.9%, the timing of the highest occupation being at 12-18 months of treatment, reaching 55% and the one with the lowest unemployment at 12 months, with 17.3%. The percentage of people with ILT falls by 13.4%. The functioning scales also detect an improvement after 2 years of treatment.

**Conclusions:**

The PEA contributes to the improvement in the global funtioning of people and their quality of life.

**Disclosure:**

No significant relationships.

